# Four-Objective Optimization of an Irreversible Magnetohydrodynamic Cycle

**DOI:** 10.3390/e24101470

**Published:** 2022-10-14

**Authors:** Qingkun Wu, Lingen Chen, Yanlin Ge, Huijun Feng

**Affiliations:** 1 Institute of Thermal Science and Power Engineering, Wuhan Institute of Technology, Wuhan 430205, China; 2 Hubei Provincial Engineering Technology Research Center of Green Chemical Equipment, Wuhan 430205, China; 3 School of Mechanical & Electrical Engineering, Wuhan Institute of Technology, Wuhan 430205, China

**Keywords:** finite time thermodynamics, NSGA-II algorithm, irreversible MHD cycle, multi-objective optimization, deviation index, performance comparison

## Abstract

Based on the existing model of an irreversible magnetohydrodynamic cycle, this paper uses finite time thermodynamic theory and multi-objective genetic algorithm (NSGA-II), introduces heat exchanger thermal conductance distribution and isentropic temperature ratio of working fluid as optimization variables, and takes power output, efficiency, ecological function, and power density as objective functions to carry out multi-objective optimization with different objective function combinations, and contrast optimization results with three decision-making approaches of LINMAP, TOPSIS, and Shannon Entropy. The results indicate that in the condition of constant gas velocity, deviation indexes are 0.1764 acquired by LINMAP and TOPSIS approaches when four-objective optimization is performed, which is less than that (0.1940) of the Shannon Entropy approach and those (0.3560, 0.7693, 0.2599, 0.1940) for four single-objective optimizations of maximum power output, efficiency, ecological function, and power density, respectively. In the condition of constant Mach number, deviation indexes are 0.1767 acquired by LINMAP and TOPSIS when four-objective optimization is performed, which is less than that (0.1950) of the Shannon Entropy approach and those (0.3600, 0.7630, 0.2637, 0.1949) for four single-objective optimizations, respectively. This indicates that the multi-objective optimization result is preferable to any single-objective optimization result.

## 1. Introduction

Finite time thermodynamic (FTT) theory has been widely used in various heat engine cycles and has made great progress [1,2,3,4,5,6,7,8,9,10,11,12,13,14,15,16,17,18,19,20,21,22,23,24,25,26,27,28,29,30,31,32,33,34,35,36,37,38]. In addition to analyzing the power output (P) and efficiency (η) performance of common engines, FTT has also been applied to heat pumps [39,40,41,42,43,44,45,46], refrigerators [47,48,49,50,51,52,53,54,55], micro-scale cycles [56,57,58,59], chemical machines [60,61,62,63,64,65,66], etc.

Angulo-Brown [67] firstly put forward ecological function (E) and analyzed the optimal performance of the Carnot engine cycle. Yan et al. [68] made amendments on this basis. Finally, Chen et al. [69] put forward a unified definition of E according to exergy analysis. Tyagi et al. [70] analyzed the irreversible Brayton cycle based on the E optimization criterion. Moscato et al. [71] researched the P, η and entropy generation rate (σ) characteristics of irreversible Otto and Diesel cycles after optimization based on E. Fernández [72] studied the η range of quantum heat engines working under the E. Jin et al. [73] optimized the E of gas turbine waste heat recovery and recompression S-CO_2_ cycle.

Sahin et al. [74] first defined power density ($P_{d}$) as an objective function (OF) to analyze the characteristics of the reversible Joule-Brayton cycle and discovered that the engine has higher η and smaller sizes in the case of maximum $P_{d}$. Maheshwari et al. [75] researched the characteristics of radiant heat engines under the case of maximum $P_{d}$. Wang et al. [76] compared the characteristics of the Atkinson cycle in the cases of maximum P and maximum $P_{d}$. Gonca [77,78] analyzed the characteristics of the Dual-Atkinson cycle [77] and Otto cycle gasoline engine [78] in the case of actual P and actual $P_{d}$. Karakurt et al. [79] analyzed and compared the maximum $P_{d}$ of the supercritical CO_2_ Brayton cycle. Gonca and Sahin [80] researched a modified Dual cycle under the condition of maximum $P_{d}$.

With the increase of OFs, conflicts may occur when multiple OFs are optimized simultaneously. Therefore, it is necessary to coordinate multiple OFs. This paper takes P, η, E, and $P_{d}$ as OFs. P represents the amount of work done per unit of time; η indicates the utilization rate of energy; E reflects the tradeoff between P and σ; $P_{d}$ reflects the tradeoff between P and thermal engine size. When one of the OFs takes the maximum value, the other OFs may have poor performance. For example, when the P takes the maximum value, the σ of the system is also large. When the E is used as the OF, although the P is reduced to a certain extent, the σ is greatly reduced. The multi-objective optimization (MOO) is to put the four OFs in an ideal state so that the cycle can achieve better performance. As an excellent multi-objective algorithm, NSGA-II [81] has been employed to MOO by many scholars. Li et al. [82] conducted MOO on the maximum P, η, and E of the solar disk Brayton system based on NSGA II. Li et al. [83] applied RSM and NSGA-II to conduct MOO on the temperature difference, pressure drop, and maximum temperature of the small U-shaped channel cold plate containing SiO_2_ Nanofluidsm and obtained the corresponding values. Ge et al. [84] studied the organic Rankine cycle under two different conditions and solved it by NSGA-II with exergy efficiency and heat recovery efficiency as OFs. Abedinnezhad et al. [85] carried out MOO of irreversible Dual-Miller cycle with η, ecological coefficient of performance and E as OFs. Yusuf et al. [86] used NSGA-II to optimize some parameters of the centralized photovoltaic thermoelectric hybrid system. Based on NSGA II, Xiao et al. [87] proposed a steam power system design and optimization strategy considering pollutant emission reduction technology to obtain the balance between environmental and economic objectives. Xu et al. [88] used NSGA-II to conduct MOO on four objectives for the Stirling heat engine considering various losses. Zang et al. [89] used the FTT to conduct thermodynamic analysis of the irreversible porous media cycle and utilized NSGA-II to conduct MOO of four objectives: dimensionless P($\bar{P}$), η, dimensionless E($\bar{E}$), and dimensionless $P_{d}$(${\bar{P}}_{d}$).

As a new type of cycle, the magnetohydrodynamic (MHD) cycle has been widely concerned because of its high efficiency and low pollution. The MHD generator allows the high-speed flow of ions to cut the magnetic induction line to generate current, so it is also called plasma power generation technology. At present, the research on MHD power generation technology is mainly focused on taking mineral fuel as the working fluid, while MHD power generation device with liquid metal as the working fluid is studied as the backup device of space power, and the capacity of the largest MHD generator has exceeded 32,000 kW. With the development of controlled thermonuclear reaction research, fusion reactive androgen MHD power generation devices may become the main form of the new central power station. There are different gas conditions in the MHD generator; therefore the two conditions of constant gas velocity (CGV) and constant Mach number (GMN) need to be discussed. FTT has also been applied to study the performances of MHD cycles. Aydin et al. [90] derived the $\bar{P}$ and η of the irreversible MHD cycle, but the loss of the compressor was ignored and only the loss of the generator was considered. Sahin et al. [91] studied the η of irreversible MHD cycles at maximum $P_{d}$. Assad [92,93] established an irreversible MHD cycle with constant temperature heat sources and studied the $\bar{P}$ and η of the cycle. Chen et al. [94] established an irreversible MHD cycle with variable temperature heat reservoirs and studied the influence of relevant parameters on $\bar{P}$ and η. Chen et al. [95] structured a regenerative MHD cycle and studied the influence of several main irreversibilities on the thermodynamic characteristic of the cycle. Wu et al. [96] performed MOO for an endoreversible MHD cycle with OFs of $\bar{P}$, η, $\bar{E}$, and efficient power.

Based on the work of Ref. [96], this paper will conduct MOO for an irreversible MHD cycle with both heat transfer loss and internal loss by NSGA-II (compared with the results of endoreversible MHD cycle [96], the results in this paper have a quantitative change). Heat exchanger (HEX), thermal conductance distribution (u), and isentropic temperature ratio (x) of working gas will be selected as optimization variables, and $\bar{P}$, η, $\bar{E}$, and ${\bar{P}}_{d}$ will be taken as OFs. Through the decision-making approaches of LINMAP [97], TOPSIS [98,99], and Shannon Entropy [100], the results of optimization with different OF combinations will be acquired, the deviation index (D) [101] will be contrasted, and then the optimal scheme with the minimum D will be acquired. The major advances of this paper are the introduction of internal loss in the cycle model and the introduction of OF ${\bar{P}}_{d}$, which replaces efficient power.

## 2. Cycle Model

**Figure 1:**
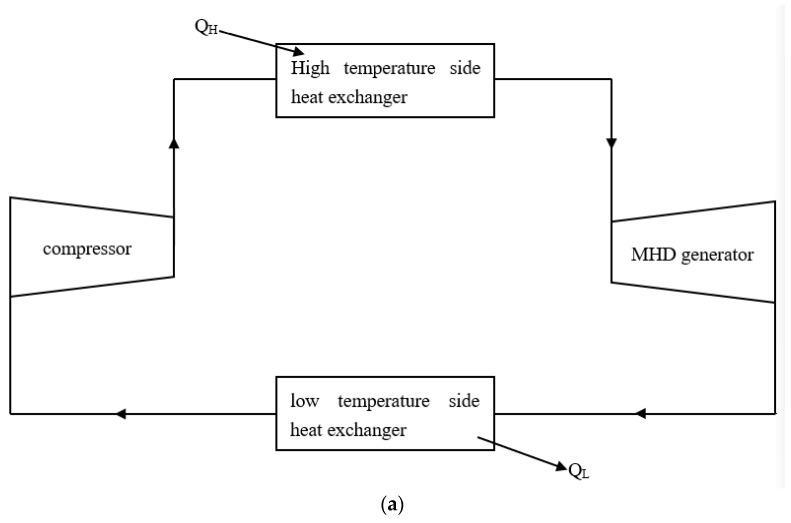

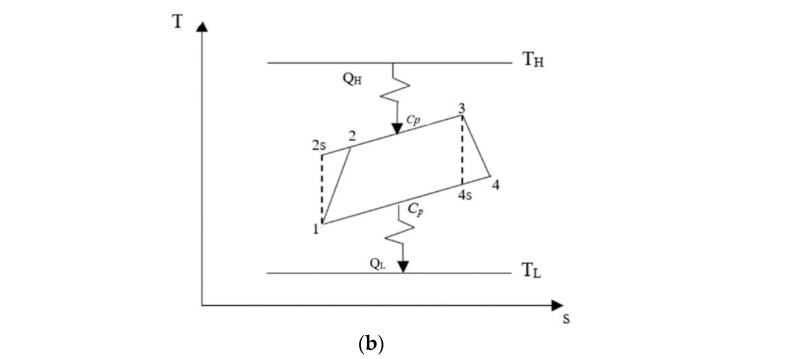
The MHD cycle model. (**a**) Cycle layout. (**b**) T−s diagram.

Figure 1 shows an MHD cycle layout and T−s diagrams. In Figure 1b, where 1→2 is the irreversible compression process in the compressor, 2→3 is the isobaric heat absorption process at the high-temperature side, 3→4 is the irreversible expansion process in the MHD generator, and 4→1 is the isobaric heat release process at the low temperature side. Processes 1→2s and 3→4s are isentropic compression and expansion processes. The circulating working gas is assumed to be an ideal gas and has a constant thermal capacitance rate $C_{wf}$.

The heat absorption rate ($Q_{H}$) and the heat release rate ($Q_{L}$) of the cycle are:

(1) $$Q_{H}=C_{wf}E_{H}(T_{H}-T_{2})=C_{wf}(T_{3}-T_{2})$$

(2) $$Q_{L}=C_{wf}E_{L}(T_{4}-T_{L})=C_{wf}(T_{4}-T_{1})$$

where $E_{H}$ and $E_{L}$ are the effectivenesses of the HEXs on t high-temperature and low-temperature sides, and $E_{H}=1-e^{-(U_{H}/C_{wf})}$, $E_{L}=1-e^{-(U_{L}/C_{wf})}$; $U_{H}$ and $U_{L}$ are the thermal conductance of the HEXs on the high-temperature and low-temperature sides.

When the total thermal conductance of the HEXs is constant, that is, $U_{H}+U_{L}=U_{T}$, the thermal conductance distribution is defined as $u=U_{H}/U_{T}$, then, there are

(3) $$U_{H}=uU_{T}$$

(4) $$U_{L}=(1-u)U_{T}$$

The P, η, E, and $P_{d}$ are expressed as

(5) $$P=Q_{H}-Q_{L}$$

(6) $$\eta =1-\frac{Q_{L}}{Q_{H}}$$

(7) $$E=P-T_{0}\sigma$$

(8) $$P_{d}=\frac{P}{V_{4}}$$

where $T_{0}$ is the surrounding temperature, and σ is the entropy generation rate:

(9) $$\sigma =\frac{Q_{L}}{T_{L}}-\frac{Q_{H}}{T_{H}}$$

where $V_{4}$ is the maximum specific volume at the generator outlet. Since the specific volume $V_{1}$ and temperature $T_{1}$ at the compressor inlet are known and the gas is an ideal one, $V_{4}$ can be expressed as

(10) $$V_{4}=\frac{T_{4}}{T_{1}}V_{1}=\frac{T_{4}}{T_{2}}\frac{T_{2}}{T_{1}}V_{1}$$

The momentum and energy equations in the MHD generator for one-dimensional steady-state flow are:

(11) ρv(dv/dZ)+dp/dZ=−JB

(12) $$\rho v[d(0.5v^{2}+h)/dZ]=-Je$$

where ρ, v, h, and p are the gas density, velocity, enthalpy, and pressure, Z is the axial direction of the generator, J is the electric current density, B is the magnetic field, and e is the electric field.

The MHD generator efficiency and compressor efficiency are expressed as

(13) $$\eta_{e}=Je/(JvB)=e/(vB)$$

(14) $$\eta_{c}=(T_{2S}-T_{1})/(T_{2}-T_{1})$$

### 2.1. Constant Gas Velocity

For the condition of CGV, it can be obtained from Equations (11)–(13):

(15) $$(\eta_{e}/k)(k-1)\;dp/p=dT/T$$

where k is the specific heat ratio of the working gas.

The x of the compressor is defined as

(16) $$x=\frac{T_{2S}}{T_{1}}={\left(\frac{p_{2}}{p_{1}}\right)}^{(k-1)/k}$$

Integrating Equations (15) and (16) yields:

(17) $$\frac{T_{3}}{T_{4}}={\left(\frac{p_{2}}{p_{1}}\right)}^{\eta_{e(k-1)/k}}=x^{\eta_{e}}$$

From the Equations (14) and (16) yields

(18) $$\frac{T_{1}}{T_{2}}=\frac{\eta_{c}}{x-1+\eta_{c}}$$

According to Equations (1), (2), (17), and (18), there are

(19) $$T_{2}=\frac{x^{\eta_{e}}E_{L}T_{L}(x-1+\eta_{c})-(E_{L}-1)E_{H}T_{H}(x-1+\eta_{c})}{\eta_{c}x^{\eta_{e}}+(E_{H}+E_{L}-E_{H}E_{L}-1)(x-1+\eta_{c})}$$

(20) $$T_{4}=\frac{\eta_{c}E_{H}T_{H}+(1-E_{H})E_{L}T_{L}(x-1+\eta_{c})}{\eta_{c}x^{\eta_{e}}+(E_{H}+E_{L}-E_{H}E_{L}-1)(x-1+\eta_{c})}$$

Integrating Equations (10), (18), (19), and (20), the $V_{4}$ can be obtained as

(21) $$V_{4}=\frac{\eta_{c}E_{H}T_{H}+(x-1+\eta_{c})(E_{L}T_{L}-E_{H}E_{L}T_{L})}{\eta_{c}x^{\eta_{e}}E_{L}T_{L}-\eta_{c}(E_{H}E_{L}T_{H}-E_{H}T_{H})}V_{1}$$

Integrating Equations (1), (2), (19), and (20), the $\bar{P}$ and η can be obtained as

(22) $${\bar{P}}_{v}=\frac{P}{C_{wf}T_{L}}=\frac{\begin{array}{l} \eta_{c}x^{\eta_{e}}E_{H}\tau +(x-1+\eta_{c})(E_{H}E_{L}\tau -E_{H}\tau -E_{L}+E_{H}E_{L})\\ -x^{\eta_{e}}(x-1+\eta_{c})E_{H}E_{L}-\eta_{c}E_{H}E_{L}\tau +\eta_{c}x^{\eta_{e}}E_{L} \end{array}}{\eta_{c}x^{\eta_{e}}+(x-1+\eta_{c})(E_{H}+E_{L}-E_{H}E_{L}-1)}$$

(23) $$\eta_{v}=\frac{\begin{array}{l} x^{\eta_{e}}\eta_{c}E_{H}\tau +(x-1+\eta_{c})(E_{H}E_{L}\tau +E_{H}E_{L}-E_{H}\tau -E_{L})\\ -(x-1+\eta_{c})x^{\eta_{e}}E_{H}E_{L}-\eta_{c}E_{H}E_{L}\tau +x^{\eta_{e}}\eta_{c}E_{L} \end{array}}{x^{\eta_{e}}\eta_{c}E_{H}\tau +E_{H}\tau (x-1+\eta_{c})(E_{L}-1)-(x-1+\eta_{c})x^{\eta_{e}}E_{H}E_{L}}$$

where $\tau =T_{H}/T_{L}$ is the temperature ratio of the cycle heat reservoirs.

According to Equations (1), (2), (7), and (9), the $\bar{E}=E/(C_{wf}T_{L})$ can be obtained as

(24) $${\bar{E}}_{v}=\frac{\begin{array}{l} \eta_{c}x^{\eta_{e}}(E_{H}\tau +E_{L})+(x-1+\eta_{c})(E_{H}E_{L}\tau +E_{H}E_{L}-E_{H}\tau -E_{L}-x^{\eta_{e}}E_{H}E_{L})-\eta_{c}E_{H}E_{L}\tau \\ -\frac{T_{0}}{T_{L}}[\eta_{c}E_{H}E_{L}\tau -\eta_{c}x^{\eta_{e}}(E_{H}+E_{L})+(x-1+\eta_{c})(x^{\eta_{e}}E_{H}E_{L}\tau^{-1}+2E_{H}E_{L}-E_{H}-E_{L})] \end{array}}{\eta_{c}x^{\eta_{e}}+(x-1+\eta_{c})(1-E_{H})(E_{L}-1)}$$

From Equations (1), (2), (8), and (21), the ${\bar{P}}_{d}=P_{d}/(C_{wf}T_{L}/V_{1})$ of the cycle can be obtained as

(25) $${\bar{P}}_{d_{v}}=\frac{\begin{array}{l} \left[\eta_{c}x^{\eta_{e}}E_{H}\tau +\eta_{c}x^{\eta_{e}}E_{L}+(x-1+\eta_{c})(E_{H}E_{L}\tau -E_{H}\tau -E_{L}+E_{H}E_{L}\right)\\ -x^{\eta_{e}}(x-1+\eta_{c})E_{H}E_{L}-\eta_{c}E_{H}E_{L}\tau ][\eta_{c}x^{\eta_{e}}E_{L}-\eta_{c}(E_{H}E_{L}-E_{H})\tau ] \end{array}}{\left[\eta_{c}x^{\eta_{e}}+(x-1+\eta_{c})(E_{H}+E_{L}-E_{H}E_{L}-1)][\eta_{c}E_{H}\tau +(x-1+\eta_{c})(E_{L}-E_{H}E_{L})\right]}$$

### 2.2. Constant Mach Number

For the condition of CGV, it can be obtained from Equations (11)–(13):

(26) $$(\eta_{e}/k)(k-1)dp/p=[0.5(1-\eta_{e})(k-1)M^{2}+1]dT/T$$

Integrating Equations (16) and (26) yields

(27) $$\frac{T_{3}}{T_{4}}=x^{\alpha }$$

where $\alpha =\eta_{e}/[0.5(1-\eta_{e})(k-1)M^{2}+1]$.

The $\bar{P}$, η, $\bar{E}$, and ${\bar{P}}_{d}$ can be obtained by comparing Equations (17) and (27)

(28) $${\bar{P}}_{M}=\frac{\begin{array}{l} \eta_{c}x^{\alpha }E_{H}\tau +(x-1+\eta_{c})(E_{H}E_{L}\tau +E_{H}E_{L}-E_{H}\tau -E_{L})\\ -(x-1+\eta_{c})x^{\alpha }E_{H}E_{L}-\eta_{c}E_{H}E_{L}\tau +\eta_{c}x^{\alpha }E_{L} \end{array}}{\eta_{c}x^{\alpha }+(x-1+\eta_{c})(1-E_{H})(E_{L}-1)}$$

(29) $$\eta_{M}=\frac{\begin{array}{l} \eta_{c}x^{\alpha }E_{H}\tau +(x-1+\eta_{c})(E_{H}E_{L}\tau +E_{H}E_{L}-E_{H}\tau -E_{L})\\ -(x-1+\eta_{c})x^{\alpha }E_{H}E_{L}-\eta_{c}E_{H}E_{L}\tau +\eta_{c}x^{\alpha }E_{L} \end{array}}{\eta_{c}x^{\alpha }E_{H}\tau +(x-1+\eta_{c})(E_{H}E_{L}\tau -E_{H}\tau )-(x-1+\eta_{c})x^{\alpha }E_{H}E_{L}}$$

(30) $${\bar{E}}_{M}=\frac{\begin{array}{l} \eta_{c}x^{\alpha }(E_{H}\tau +E_{L})+(x-1+\eta_{c})(E_{H}E_{L}\tau +E_{H}E_{L}-E_{H}\tau -E_{L}-x^{\alpha }E_{H}E_{L})-\eta_{c}E_{H}E_{L}\tau \\ -\frac{T_{0}}{T_{L}}[\eta_{c}E_{H}E_{L}\tau -\eta_{c}x^{\alpha }(E_{H}+E_{L})+(x-1+\eta_{c})(E_{H}+E_{L}-2E_{H}E_{L}+x^{\alpha }E_{H}E_{L}\tau^{-1})] \end{array}}{\eta_{c}x^{\alpha }+(x-1+\eta_{c})(1-E_{H})(E_{L}-1)}$$

(31) $${\bar{P}}_{d_{M}}=\frac{\begin{array}{l} \left[\eta_{c}x^{\alpha }E_{H}\tau +\eta_{c}x^{\alpha }E_{L}+(x-1+\eta_{c})(E_{H}E_{L}\tau -E_{H}\tau -E_{L}+E_{H}E_{L}\right)\\ -x^{\alpha }(x-1+\eta_{c})E_{H}E_{L}-\eta_{c}E_{H}E_{L}\tau ][\eta_{c}x^{\alpha }E_{L}-\eta_{c}(E_{H}E_{L}-E_{H})\tau ] \end{array}}{\left[\eta_{c}x^{\alpha }+(x-1+\eta_{c})(E_{H}+E_{L}-E_{H}E_{L}-1)][\eta_{c}E_{H}\tau +(x-1+\eta_{c})(E_{L}-E_{H}E_{L})\right]}$$

## 3. Multi-Objective Optimizations

**Figure 2:**
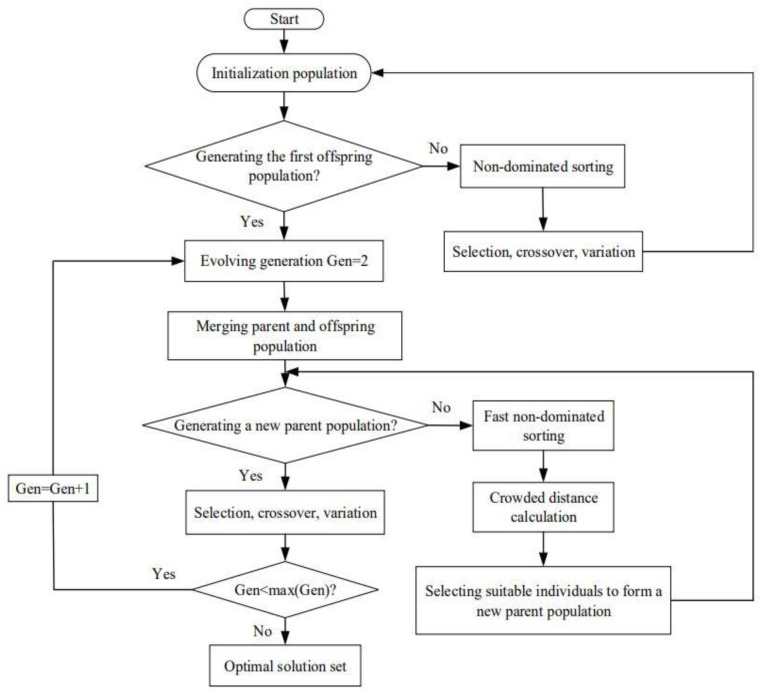
Flow chart of genetic algorithm.

MOO does not mean that each OF reaches the maximum value. Its essence is to balance the advantages and disadvantages of each OFs through NSGA-II to achieve the best compromise of different OFs and obtain a series of feasible solutions. It is also called the Pareto frontier. Figure 2 is an algorithm flowchart of NSGA-II. The NSGA-II algorithm has the advantages of fast running speed and good convergence of solution sets. It not only reduces the computational complexity but also retains all the best individuals, thus improving the accuracy of the optimization results. Its procedure is as follows: first, initialize the population and set the evolution algebra as one; second, non-dominated sorting and selection, Gaussian crossing, and mutation are carried out on the initial population to generate the first-generation sub-population and add one to the evolution algebra, and then the parent population and the child population are merged; third, calculate the objective function of individuals in the new population, and generate a new parent population by performing fast non-dominated sorting, computing crowding, elite strategy, and other operations at the same time, and then perform selection, crossover, and mutation operations on the generated parent population to generate a child population; finally, judge whether the evolution algebra is equal to the maximum evolution algebra. If not, the evolution algebra will be added and returned to the third step. Otherwise, the algorithm will end. After the results of different OF combinations are acquired, the D are compared through three approaches.

There is no good or bad between the three decision-making approaches. They have their own priorities. In actual operation, the decision approach can be selected according to these needs. For the LINMAP approach, the point with the shortest space distance from the positive ideal point is taken as the required optimal point. By definition, the Euclidean distance is

(32) $$ED_{i+}=\sqrt{\sum_{j=1}^{m}{\left(f_{ij}-f_{j}{}^{positive}\right)}^{2}}$$

(33) $$ED_{i-}=\sqrt{\sum_{j=1}^{m}{\left(f_{ij}-f_{j}{}^{negative}\right)}^{2}}$$

where i∈[1,n] is the *i*-th point (the *i*-th optimal solution) in the Pareto frontier, j∈[1,m] is the *j*-th objective function, $f_{ij}$ is the value of the *j*-th objective function of the *i*-th optimal solution, $f_{j}{}^{positive}$ is the value of the *j*-th objective function of the positive ideal point, and $f_{j}{}^{negative}$ is the value of the *j*-th objective function of the negative ideal point. Then the best feasible solution $i_{opt}$ obtained by LINMAP approach is

(34) $$i_{opt}=i\in \min(ED_{i+})$$

For the TOPSIS approach, the point with the largest space distance from negative ideal points and the shortest space distance from positive ideal points is taken as the optimal point. According to Equations (32) and (33), the best feasible solution $i_{opt}$ obtained by the TOPSIS approach is

(35) $$i_{opt}=i\in \max(\frac{ED_{i-}}{ED_{i+}+ED_{i-}})$$

For the Shannon Entropy approach, the point is taken as the required optimal point when the last OF is optimal. The best feasible solution $i_{opt}$ obtained by the Shannon Entropy approach is

(36) $$i_{opt}=i\in \max(P_{ij}\times W_{j})$$

where

(37) $$P_{ij}=\frac{f_{ij}}{\sum_{i=1}^{n}f_{ij}}$$

(38) $$SEj=-\frac{1}{\ln n}\sum_{i=1}^{n}P_{ij}\ln Pi_{j}$$

(39) $$W_{j}=\frac{\left(1-SE_{j}\right)}{\sum_{j=1}^{m}\left(1-SE_{j}\right)}$$

Based on the above results, the D is

(40) $$D=\frac{\sqrt{\sum_{j=1}^{m}{\left(G_{j}-G_{j}^{\mathrm{positive}}\right)}^{2}}}{\sqrt{\sum_{j=1}^{m}{\left(G_{j}-G_{j}^{\mathrm{positive}}\right)}^{2}}+\sqrt{\sum_{j=1}^{m}{\left(G_{j}-G_{j}^{\mathrm{negative}}\right)}^{2}}}$$

where $G_{j}$ is the *j*-th optimization objective, $G_{j}^{\mathrm{positive}}$ is the *j*-th optimization objective of the positive ideal point, and $G_{j}^{\mathrm{negative}}$ is the *j*-th optimization objective of the negative ideal point.

For the Shannon Entropy approach, this paper settles the D obtained by solving each OF as the last optimization objective, and then selects the scheme with the smallest D.

The parameter values in the calculations are as follows: $\eta_{c}=\eta_{e}=0.95$, M=0.5, τ=5, k=1.4, $C_{wf}=1\;\mathrm{kW/W}$, $U_{T}=5\;\mathrm{kW/W}$, $T_{0}=300\;K$, $T_{L}=300\;K$.

### 3.1. Constant Gas Velocity

Table 1 is the numerical results of optimizations. The results show that the D are 0.1764 acquired by LINMAP and TOPSIS when the MOO is performed on $\bar{P}-\eta -\bar{E}-{\bar{P}}_{d}$, while D are 0.3560, 0.7693, 0.2599, and 0.1940, respectively, for four single-objective optimizations of maximum $\bar{P}$, η, $\bar{E}$, and ${\bar{P}}_{d}$. It shows that the results of MOO are preferable to those of any single objective optimizations, and MOO can better consider different optimization objectives by selecting appropriate decision-making approaches. For MOO of $\bar{P}-\eta$, the D acquired by the TOPSIS is 0.1600, which is smaller than those acquired by the single objective optimizations and the combination optimizations of other OFs, and the scheme is the most reasonable.

**Figure 3:**
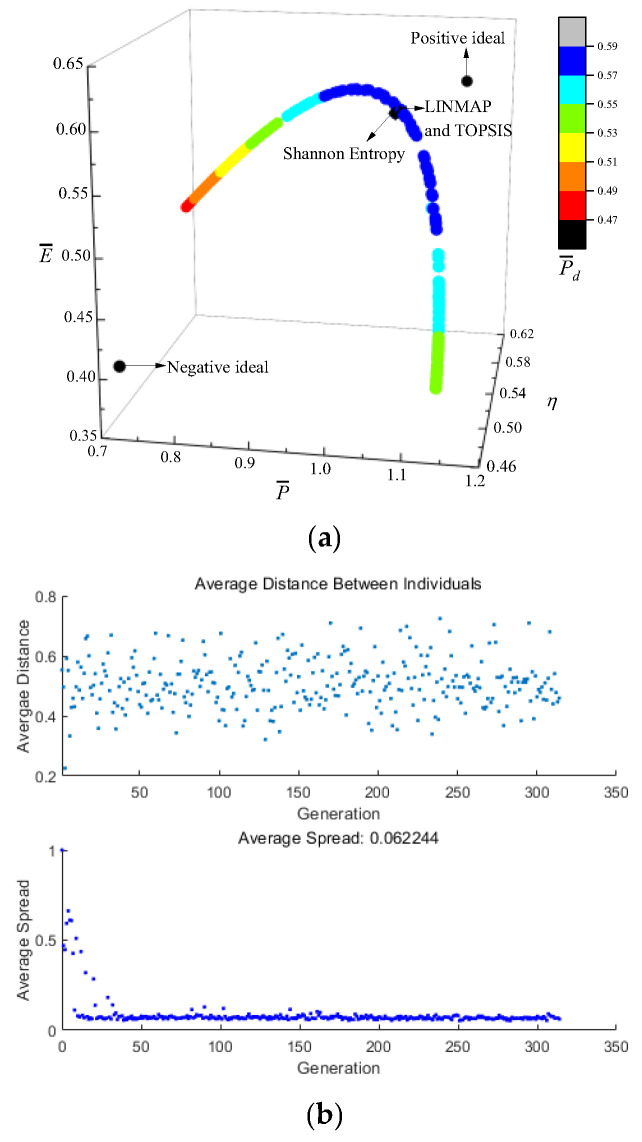
Results of quadru-objective optimization. (**a**) Pareto frontier of $\bar{P}-\eta -\bar{E}-{\bar{P}}_{d}$. (**b**) Average spread and generation number of $\bar{P}-\eta -\bar{E}-{\bar{P}}_{d}$.

Figure 3 shows the results of $\bar{P}-\eta -\bar{E}-{\bar{P}}_{d}$ optimization. In Figure 3a, the coordinate axis represents $\bar{P}$, η, and $\bar{E}$ respectively, and ${\bar{P}}_{d}$ is expressed by a color gradient. As $\bar{P}$ raises, η reduces, $\bar{E}$ and ${\bar{P}}_{d}$ first raise and then reduce. Figure 3b is the average distance generation and average spread generation and converges in the 315th generation. According to Table 1, for single objective optimization, the D is the minimum when ${\bar{P}}_{d}$ is the maximum. Compared with the single objective optimization result when ${\bar{P}}_{d}$ is maximum, ${\bar{P}}_{d}$ decreases from 0.5899 to 0.5871, reducing by 0.47%, but $\bar{P}$ increases from 1.0475 to 1.0587, increasing by 1.07%, η decreases from 0.5552 to 0.5535, reducing by 0.31%, and $\bar{E}$ increases from 0.5857 to 0.5873, increasing by 0.27%. The D acquired by the TOPSIS and LINMAP are 0.1764, which is less than that by the Shannon Entropy, and this scheme is more ideal.

**Figure 4:**
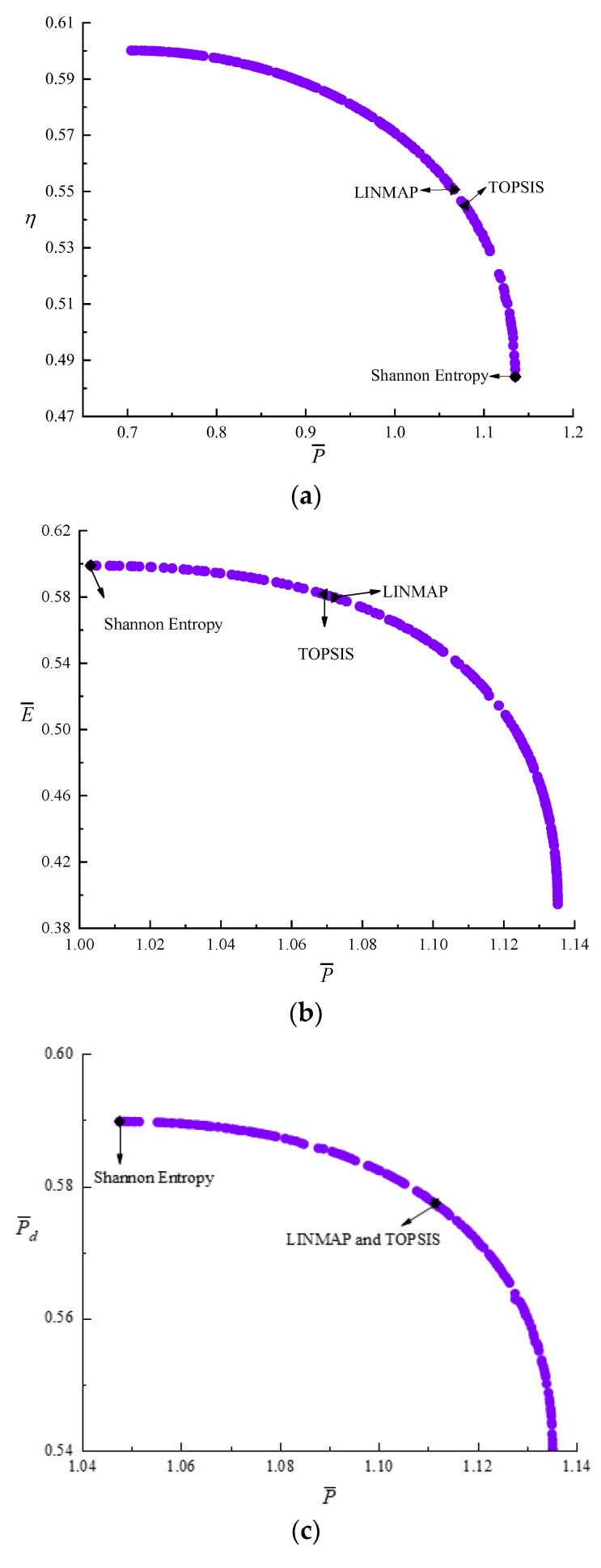

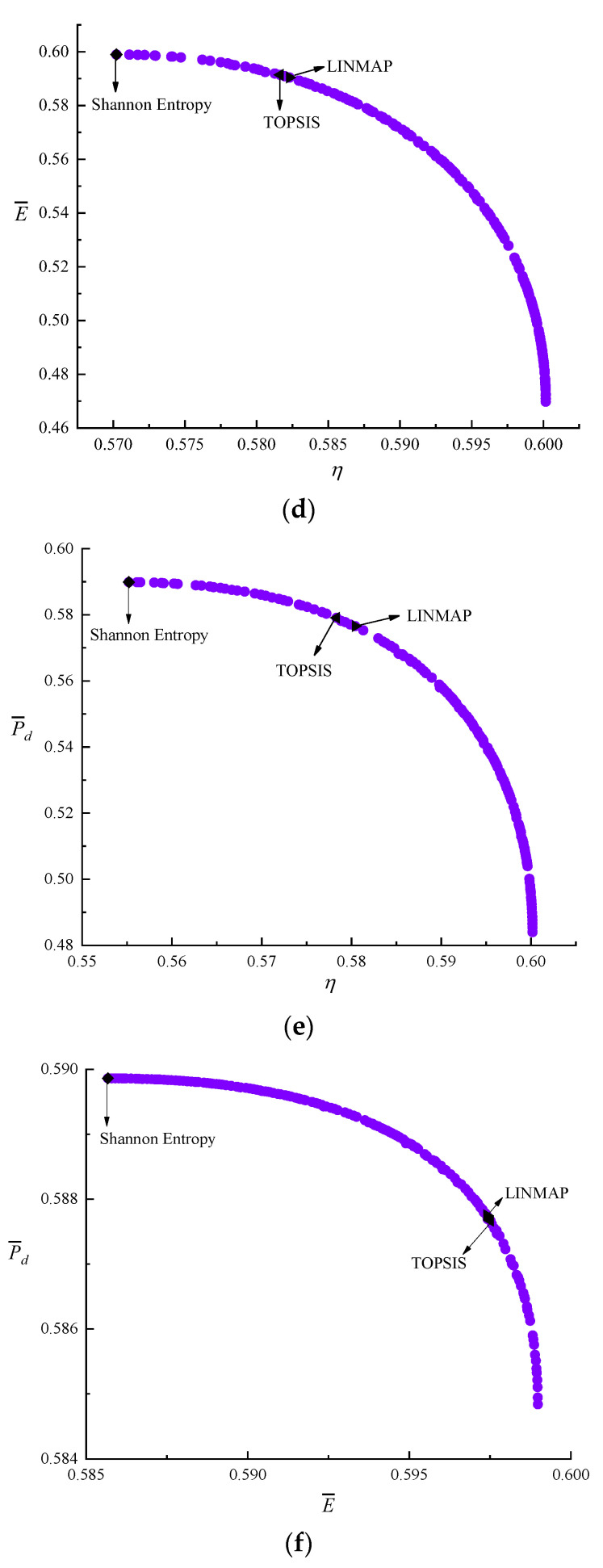

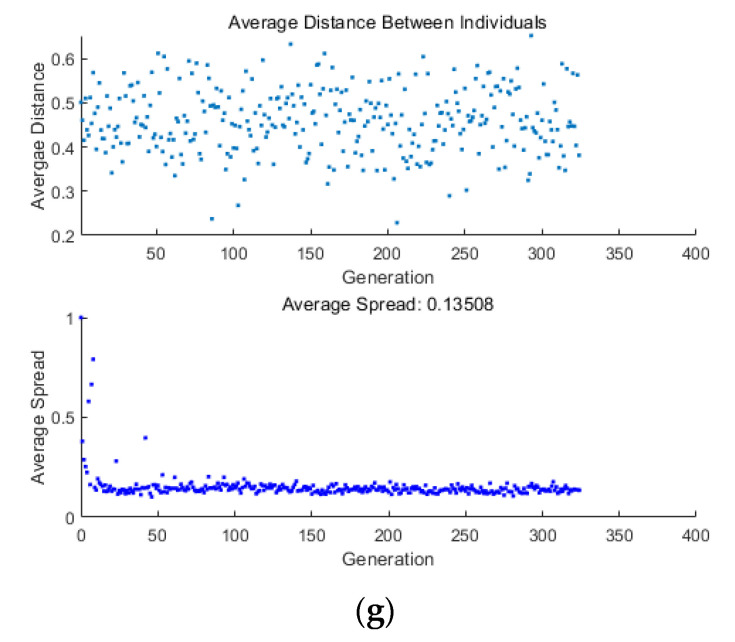
Results of bi-objective optimization. (**a**) Pareto frontier of $\bar{P}-\eta$. (**b**) Pareto frontier of $\bar{P}-\bar{E}$. (**c**) Pareto frontier of $\bar{P}-{\bar{P}}_{d}$. (**d**) Pareto frontier of $\eta -\bar{E}$. (**e**) Pareto frontier of $\eta -{\bar{P}}_{d}$. (**f**) Pareto frontier of $\bar{E}-{\bar{P}}_{d}$. (**g**) Average spread and generation number of $\bar{P}-\eta$.

Figure 4 shows the results of bi-objective optimizations. According to Figure 4a–f, as $\bar{P}$ raises, η, $\bar{E}$, and ${\bar{P}}_{d}$ all reduce. As η raises, $\bar{E}$ and ${\bar{P}}_{d}$ reduce. As $\bar{E}$ raises, ${\bar{P}}_{d}$ reduces. According to Table 1, the D acquired by the LINMAP is less than those by the other two approaches when $\bar{P}$ and $\bar{E}$ are applied as the OFs. When $\bar{P}$ and η are applied as the OFs, the D acquired by TOPSIS is less than those by the other two approaches. When $\bar{E}$ and ${\bar{P}}_{d}$ or η and ${\bar{P}}_{d}$ or $\bar{P}$ and ${\bar{P}}_{d}$ or η and $\bar{E}$ are applied as the OFs, the D acquired by Shannon Entropy is less than those by the other two approaches. Figure 4g is the average distance generation and average spread generation and converges in the 325th generation when the $\bar{P}$ and η are applied as the OFs, and the D acquired by Shannon Entropy is 0.1600, which is smaller than other results. Compared with the single objective optimization result when ${\bar{P}}_{d}$ is maximum, ${\bar{P}}_{d}$ decreases from 0.5899 to 0.5859, reducing by 0.68%, but $\bar{P}$ increases from 1.0475 to 1.0794, increasing by 3.05%, η decreases from 0.5552 to 0.5450, reducing by 1.84%, and $\bar{E}$ decreases from 0.5857 to 0.5743, reducing by 1.95%. This scheme is ideal.

**Figure 5:**
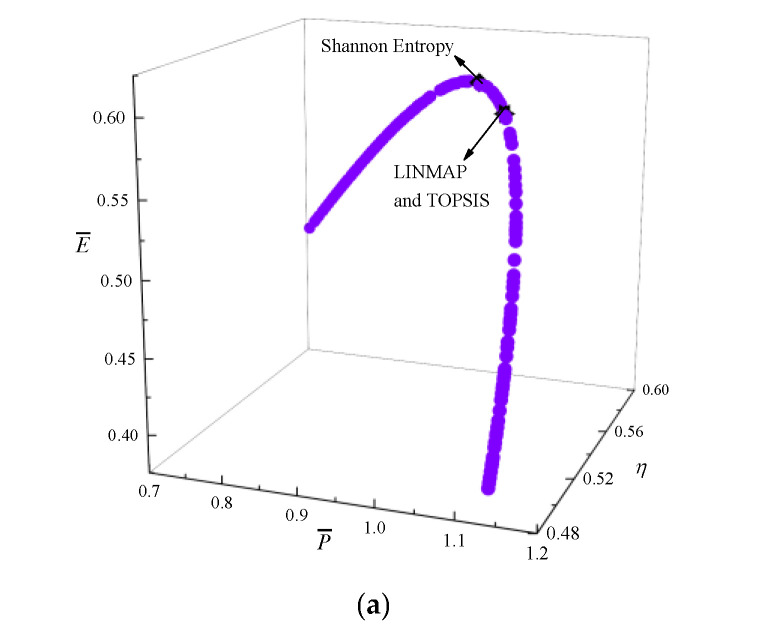

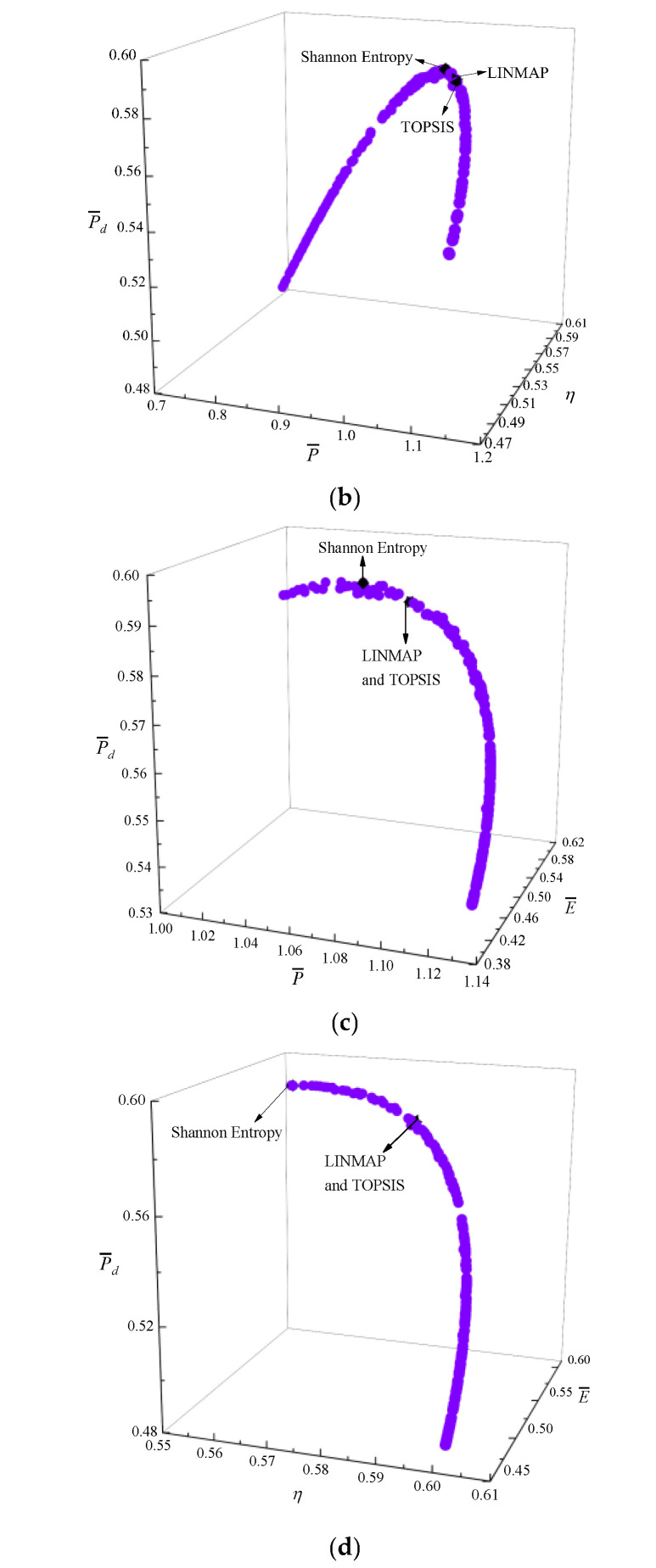

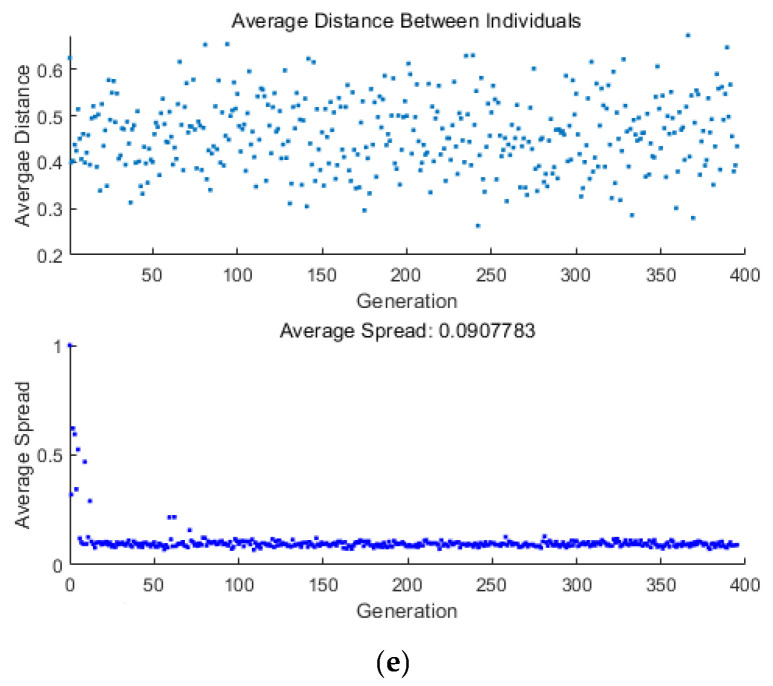
Results of bi-objective optimization. (**a**) Pareto frontier of $\bar{P}-\eta -\bar{E}$. (**b**) Pareto frontier of $\bar{P}-\eta -{\bar{P}}_{d}$. (**c**) Pareto frontier of $\bar{P}-\bar{E}-{\bar{P}}_{d}$. (**d**) Pareto frontier of $\eta -\bar{E}-{\bar{P}}_{d}$. (**e**) Average spread and generation number of $\bar{P}-\eta -{\bar{P}}_{d}$.

Figure 5 shows the results of tri-objective optimizations. In term of Figure 5a–d, as $\bar{P}$ raises, η reduces, $\bar{E}$ and ${\bar{P}}_{d}$ raise first and then reduce. As η raises, $\bar{E}$ and ${\bar{P}}_{d}$ all reduce. According to Table 1 that when $\bar{P}$, $\bar{E}$ and ${\bar{P}}_{d}$ or $\bar{P}$, η and $\bar{E}$ are applied as OFs, the Ds acquired by TOPSIS and LINMAP are equal, and less than that by the Shannon Entropy. When $\bar{P}$, η, and ${\bar{P}}_{d}$ are applied as OFs, the D acquired by TOPSIS is less than those by the other two approaches. When η, $\bar{E}$, and ${\bar{P}}_{d}$ are applied as OFs, the D acquired by Shannon Entropy is less than those by the other two approaches. Figure 5e is the average distance generation and average spread generation and converges in the 396th generation when $\bar{P}$, η, and ${\bar{P}}_{d}$ are applied as the OFs for tri-objective optimization, and the D acquired by the TOPSIS approach is 0.1624, which is smaller than other results. Compared with the single objective optimization result when ${\bar{P}}_{d}$ is maximum, ${\bar{P}}_{d}$ decreases from 0.5899 to 0.5868, reducing by 0.53%, but $\bar{P}$ increases from 1.0475 to 1.0742, increasing by 2.55%, η decreases from 0.5552 to 0.5473, reducing by 1.42%, and $\bar{E}$ decreases from 0.5857 to 0.5783, reducing by 1.26%. This scheme is ideal.

### 3.2. Constant Mach Number

Table 2 is the numerical results of optimizations. The results show that the Ds are 0.1767 acquired by LINMAP and TOPSIS when the MOO is performed on $\bar{P}-\eta -\bar{E}-{\bar{P}}_{d}$ optimization, while Ds are 0.3600, 0.7630, 0.2637, and 0.1949, respectively, for four single-objective optimizations of maximum $\bar{P}$, η, $\bar{E}$ and ${\bar{P}}_{d}$. It shows that the results of MOO are preferable. For MOO of $\bar{P}-\eta$, the D acquired by the TOPSIS is 0.1603, which is smaller than those acquired by single objective optimization and combination optimizations of other OFs, and the scheme is the most reasonable.

**Figure 6:**
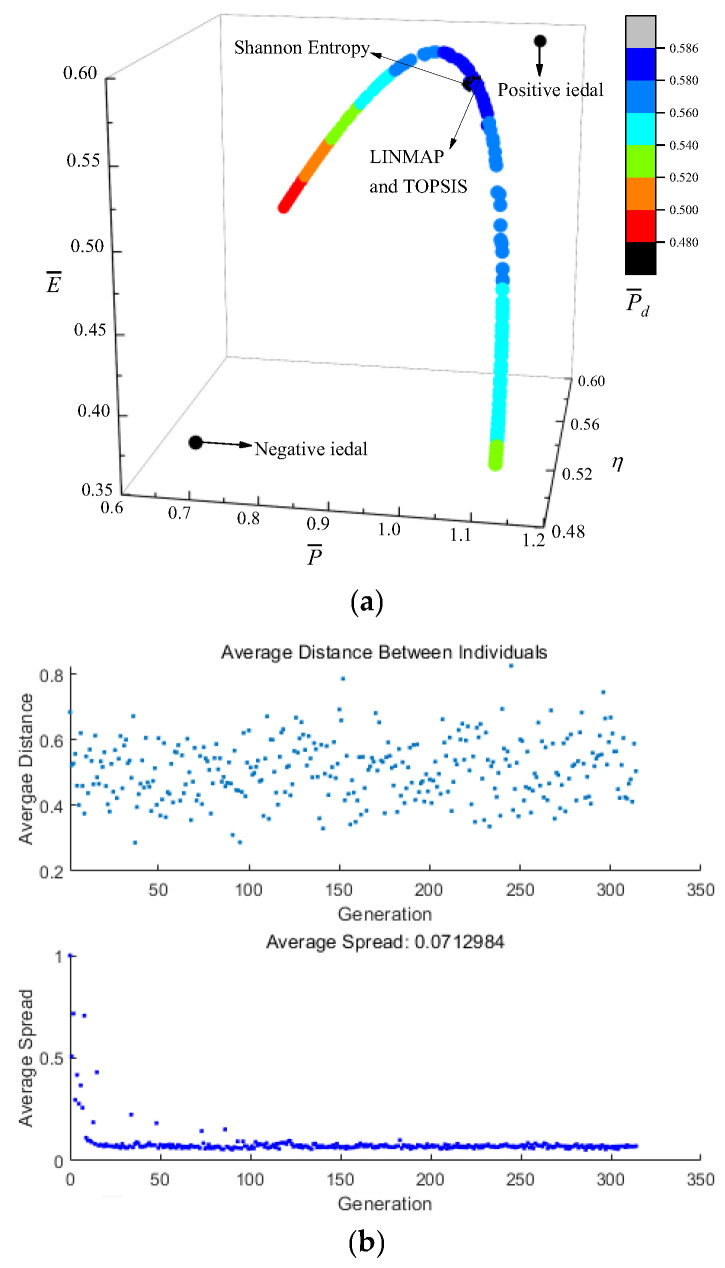
Results of quadru-objective optimization. (**a**) Pareto frontier of $\bar{P}-\eta -\bar{E}-{\bar{P}}_{d}$. (**b**) Average spread and generation number of $\bar{P}-\eta -\bar{E}-{\bar{P}}_{d}$.

Figure 6 shows the results of $\bar{P}-\eta -\bar{E}-{\bar{P}}_{d}$ optimization. In Figure 6a, as $\bar{P}$ raises, η reduces, $\bar{E}$ and ${\bar{P}}_{d}$ first raise and then reduce. Figure 6b is the average distance generation and average spread generation and converges in the 315th generation. According to Table 2, compared with the single objective optimization result when ${\bar{P}}_{d}$ is maximum, ${\bar{P}}_{d}$ decreases from 0.5859 to 0.5836, reducing by 0.39%, but $\bar{P}$ increases from 1.0440 to 1.0552, increasing by 1.07%, η decreases from 0.5524 to 0.5507, reducing by 0.31%, and $\bar{E}$ increases from 0.5759 to 0.5776, increasing by 0.30%. The Ds acquired by the TOPSIS and LINMAP are 0.1767, which is less than that by the Shannon Entropy, and this scheme is ideal.

**Figure 7:**
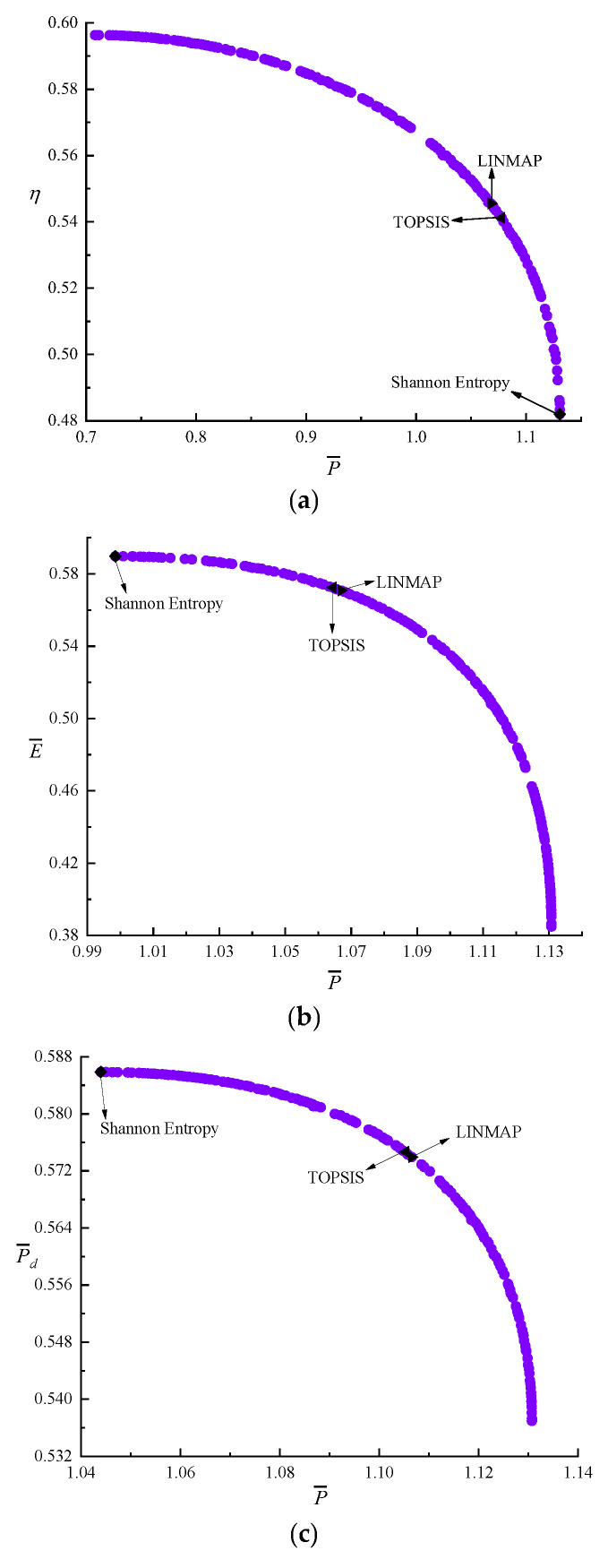

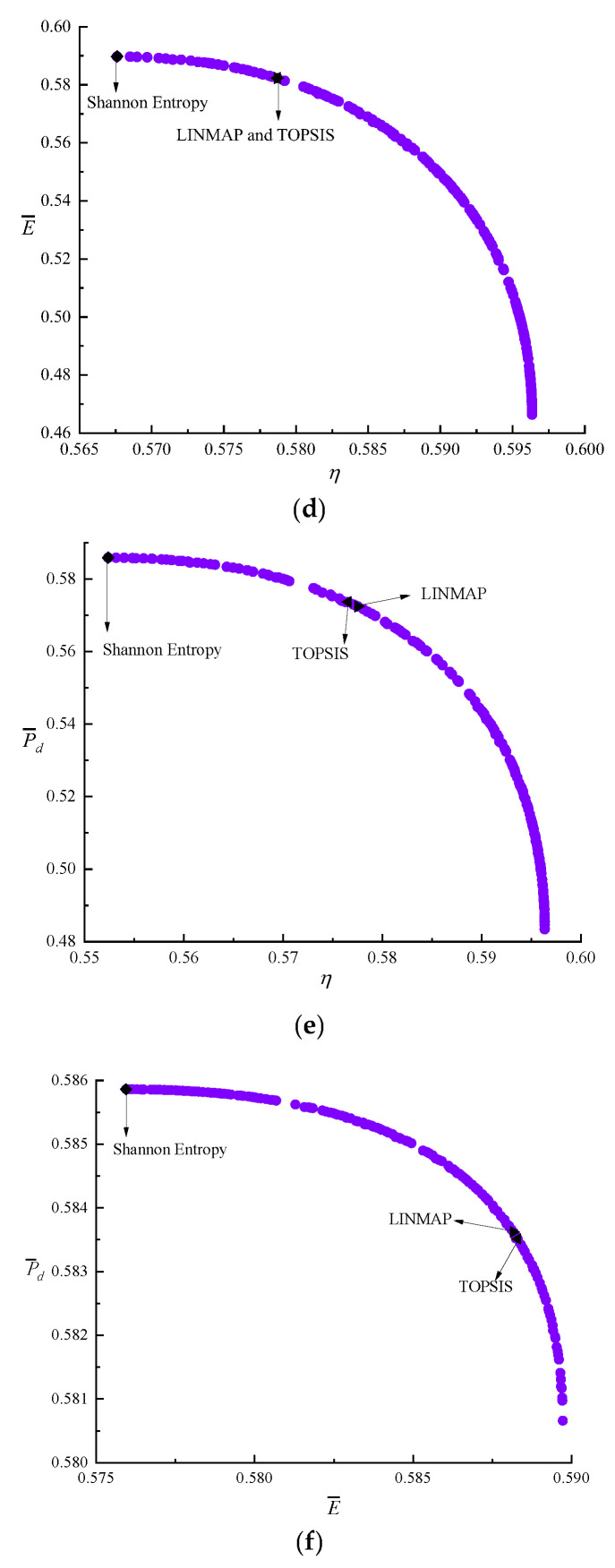

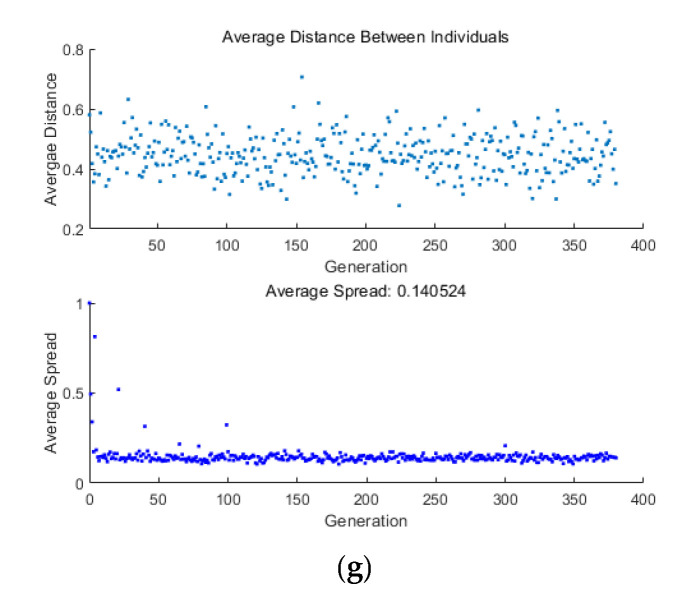
Results of bi-objective optimization. (**a**) Pareto frontier of $\bar{P}-\eta$. (**b**) Pareto frontier of $\bar{P}-\bar{E}$. (**c**) Pareto frontier of $\bar{P}-{\bar{P}}_{d}$. (**d**) Pareto frontier of $\eta -\bar{E}$. (**e**) Pareto frontier of $\eta -{\bar{P}}_{d}$. (**f**) Pareto frontier of $\bar{E}-{\bar{P}}_{d}$. (**g**) Average spread and generation number of $\bar{P}-\eta$.

Figure 7 shows the results of bi-objective optimizations. According to Figure 7a–f, as $\bar{P}$ raises, η, $\bar{E}$, and ${\bar{P}}_{d}$ all reduce. As η raises, $\bar{E}$ and ${\bar{P}}_{d}$ reduce. As $\bar{E}$ raises, ${\bar{P}}_{d}$ reduces. Form Table 2, the D acquired by the LINMAP is less than those by the other two approaches when $\bar{P}$ and ${\bar{P}}_{d}$ or $\bar{P}$ and $\bar{E}$ are applied as OFs. The D acquired by the TOPSIS is less than those by the other two approaches when $\bar{P}$ and η are applied as the OFs. When $\bar{E}$ and ${\bar{P}}_{d}$ or η and $\bar{E}$ or η and ${\bar{P}}_{d}$ are applied as the OF, the D acquired by Shannon Entropy is less than those by the other two approaches. Figure 7g is the average distance generation and average spread generation and converges in the 381th generation when the $\bar{P}$ and η are applied as the OFs, and the D acquired by TOPSIS is 0.1603, which is smaller than the other results. Compared with the single objective optimization result when ${\bar{P}}_{d}$ is maximum, ${\bar{P}}_{d}$ decreases from 0.5859 to 0.5817, reducing by 0.72%, but $\bar{P}$ increases from 1.0440 to 1.0778, increasing by 3.24%, η decreases from 0.5524 to 0.5412, reducing by 2.03%, and $\bar{E}$ decreases from 0.5759 to 0.5623, reducing by 2.36%. This scheme is ideal.

**Figure 8:**
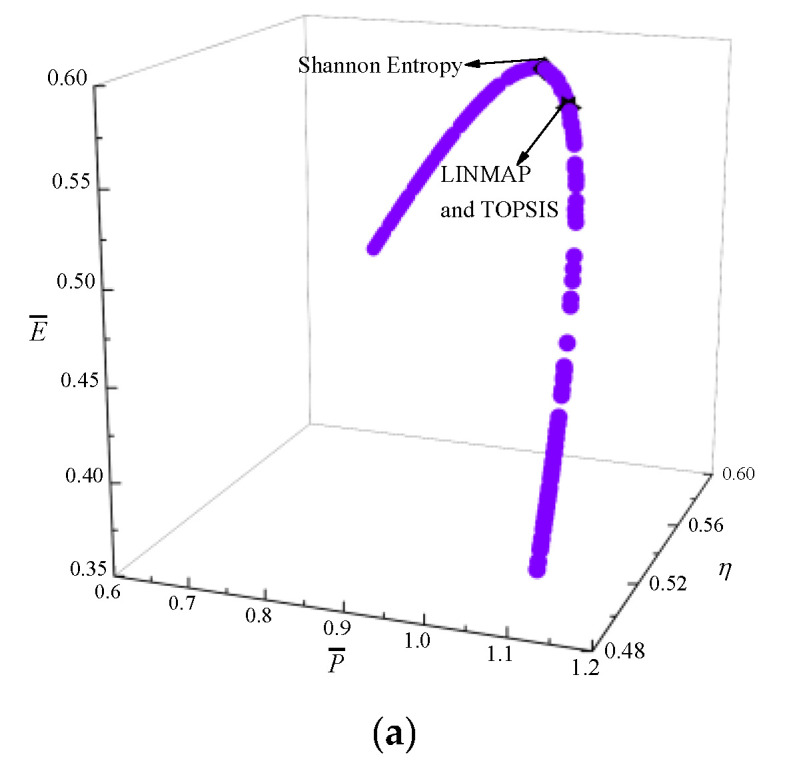

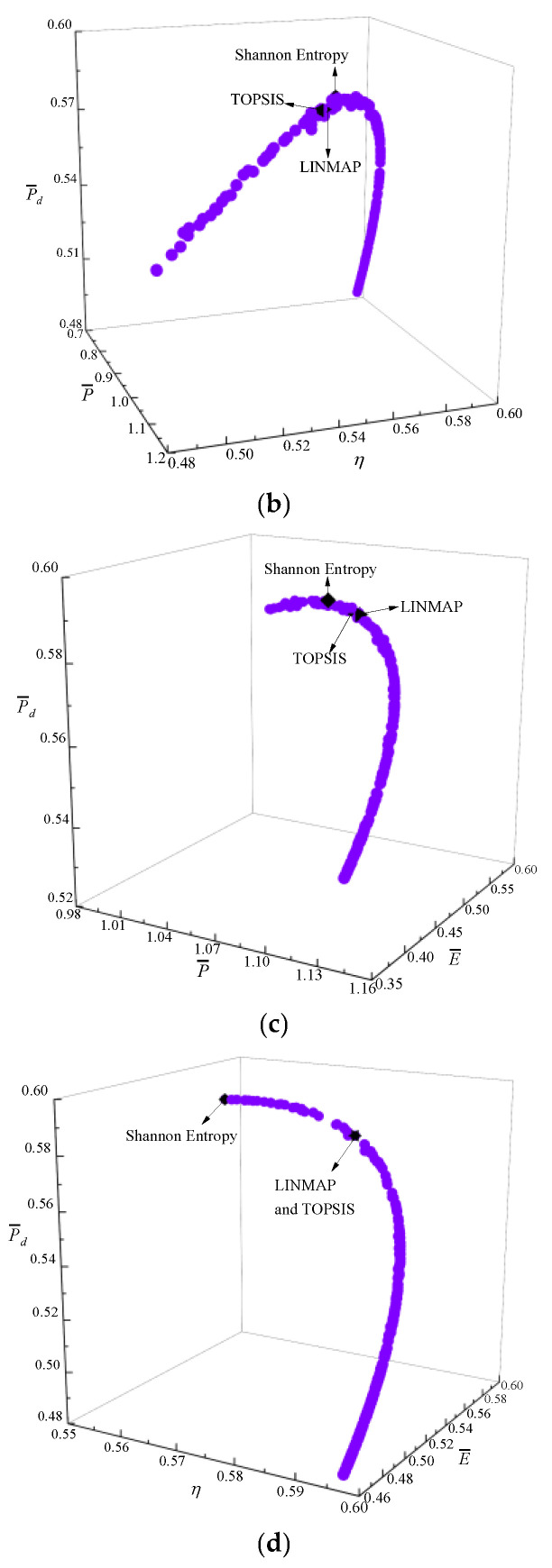

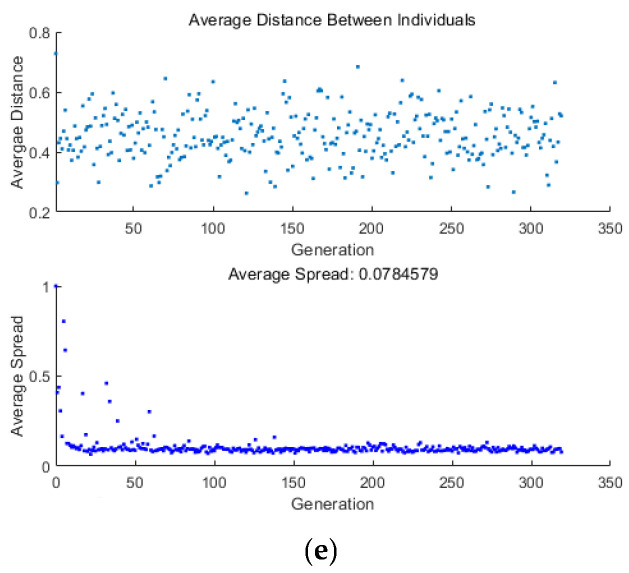
Results of bi-objective optimization. (**a**) Pareto frontier of $\bar{P}-\eta -\bar{E}$. (**b**) Pareto frontier of $\bar{P}-\eta -{\bar{P}}_{d}$. (**c**) Pareto frontier of $\bar{P}-\bar{E}-{\bar{P}}_{d}$. (**d**) Pareto frontier of $\eta -\bar{E}-{\bar{P}}_{d}$. (**e**) Average spread and generation number of $\bar{P}-\eta -{\bar{P}}_{d}$.

Figure 8 shows the results of tri-objective optimizations. According to Figure 8a–d, as $\bar{P}$ raises, η reduces, $\bar{E}$ and ${\bar{P}}_{d}$ raise first and then reduce. As η raises, $\bar{E}$ and ${\bar{P}}_{d}$ all reduce. According to Table 2 that the Ds acquired by TOPSIS and LINMAP are the equal and less than that by Shannon Entropy when $\bar{P}$, η, and $\bar{E}$ are applied as OFs. When $\bar{P}$, $\bar{E}$, and ${\bar{P}}_{d}$ are applied as OFs, the D acquired by LINMAP is less than those by the other two approaches. When $\bar{P}$, η, and ${\bar{P}}_{d}$ are applied as OFs, the D acquired by TOPSIS is less than those by the other two approaches. When η, $\bar{E}$, and ${\bar{P}}_{d}$ are applied as OFs, the D acquired by the Shannon Entropy is less than those by the other two approaches. Figure 8e is the average distance generation and average spread generation and converges in the 320th generation when the $\bar{P}$, η, and ${\bar{P}}_{d}$ are applied as the OFs, and the D acquired by TOPSIS is 0.1623, which is smaller than the other results. Compared with the single objective optimization result when ${\bar{P}}_{d}$ is maximum, ${\bar{P}}_{d}$ decreases from 0.5859 to 0.5830, reducing by 0.49%, but $\bar{P}$ increases from 1.0440 to 1.0721, increasing by 2.69%, η decreases from 0.5524 to 0.5438, reducing by 1.56%, and $\bar{E}$ decreases from 0.5759 to 0.5669, reducing by 1.56%. This scheme is ideal.

## 4. Conclusions

According to the existing irreversible MHD model with constant-temperature heat reservoirs, this paper adds internal loss and conducts the MOO of $\bar{P}$, η, $\bar{E}$, and ${\bar{P}}_{d}$. Through three decision-making approaches, the optimization results under different OF combinations are acquired. The results show that:

1. In the condition of CGV, the D acquired by TOPSIS and LINMAP are 0.1764 for MOO of $\bar{P}-\eta -\bar{E}-{\bar{P}}_{d}$, which is less than 0.3560, 0.7693, 0.2599, and 0.1940 for the four single-objective optimizations with maximum $\bar{P}$, η, $\bar{E}$, and ${\bar{P}}_{d}$, respectively. Four-objective optimization results are better.
2. In the condition of CMN, the D acquired by LINMAP and TOPSIS are 0.1767 for MOO of $\bar{P}-\eta -\bar{E}-{\bar{P}}_{d}$, which is less than 0.3600, 0.7630, 0.2637, and 0.1949 for the four single-objective optimizations with maximum $\bar{P}$, η, $\bar{E}$, and ${\bar{P}}_{d}$, respectively. Four-objective optimization results are better.
3. In the condition of CGV, when MOO is conducted on $\bar{P}-\eta$, the D is the 0.1600 acquired by TOPSIS, which is the most reasonable solution. In the condition of CMN, when MOO is conducted on $\bar{P}-\eta$, the D is the 0.1603 acquired by TOPSIS, which is the most reasonable solution. The MHD cycle has better performance in the condition of CGV.
4. Compared with single-objective optimization, MOO can better take different optimization objectives into account by choosing appropriate decision-making approaches. For the results of different objective combinations, appropriate schemes can be selected according to the actual design and operation to meet the requirements under different working conditions.
5. For the follow-up research of the MHD cycle, more variables and OFs, or the heat regenerative process, can be added so as to provide more research support for the operation of the actual MHD cycle.

## Figures and Tables

**Table 1:** Results of single-, bi-, tri-, and quadru-objective optimizations.

Optimization Objective	Decision-Making Approaches	Optimization Variables		Optimization Objectives				Deviation Index
		x	u	${\bar{P}}_{s}$	$\eta_{s}$	$\bar{E}$	${\bar{P}}_{d}$	D
Quadru-objective optimization ($\bar{P}$, η, $\bar{E}$ and ${\bar{P}}_{d}$)	LINMAP	2.6234	0.4649	1.0587	0.5535	0.5873	0.5871	0.1764
	TOPSIS	2.6234	0.4649	1.0587	0.5535	0.5873	0.5871	0.1764
	Shannon Entropy	2.6480	0.5153	1.0476	0.5552	0.5857	0.5899	0.1940
Tri-objective optimization ($\bar{P}$, η and $\bar{E}$)	LINMAP	2.6206	0.4752	1.0600	0.5531	0.5868	0.5880	0.1767
	TOPSIS	2.6206	0.4752	1.0600	0.5531	0.5868	0.5880	0.1747
	Shannon Entropy	2.7945	0.4682	1.0032	0.5702	0.5990	0.5848	0.2599
Tri-objective optimization ($\bar{P}$, η and ${\bar{P}}_{d}$)	LINMAP	2.5823	0.4800	1.0708	0.5487	0.5805	0.5875	0.1647
	TOPSIS	2.5696	0.4753	1.0742	0.5473	0.5783	0.5868	0.1624
	Shannon Entropy	2.6481	0.5153	1.0475	0.5552	0.5857	0.5899	0.1940
Tri-objective optimization ($\bar{P}$, $\bar{E}$ and ${\bar{P}}_{d}$)	LINMAP	2.5769	0.4715	1.0721	0.5482	0.5797	0.5867	0.1638
	TOPSIS	2.5769	0.4715	1.0721	0.5482	0.5797	0.5867	0.1638
	Shannon Entropy	2.6477	0.5155	1.0476	0.5552	0.5856	0.5899	0.1940
Tri-objective optimization (η, $\bar{E}$ and ${\bar{P}}_{d}$)	LINMAP	2.9151	0.4805	0.9572	0.5797	0.5933	0.5775	0.3402
	TOPSIS	2.9151	0.4805	0.9572	0.5797	0.5933	0.5775	0.3402
	Shannon Entropy	2.6480	0.5153	1.0476	0.5552	0.5857	0.5899	0.1940
Bi-objective optimization ($\bar{P}$ and η)	LINMAP	2.5983	0.4713	1.0662	0.5507	0.5835	0.5872	0.1684
	TOPSIS	2.5495	0.4743	1.0794	0.5450	0.5743	0.5859	0.1600
	Shannon Entropy	2.1467	0.4827	1.1353	0.4841	0.3945	0.5403	0.3561
Bi-objective optimization ($\bar{P}$ and $\bar{E}$)	LINMAP	2.5775	0.4760	1.0721	0.5482	0.5798	0.5871	0.1637
	TOPSIS	2.5875	0.4716	1.0692	0.5494	0.5816	0.5870	0.1659
	Shannon Entropy	2.7945	0.4683	1.0032	0.5702	0.5990	0.5848	0.2599
Bi-objective optimization ($\bar{P}$ and ${\bar{P}}_{d}$)	LINMAP	2.3947	0.5002	1.1115	0.5244	0.5272	0.5775	0.1967
	TOPSIS	2.3947	0.5002	1.1115	0.5244	0.5272	0.5775	0.1967
	Shannon Entropy	0.6481	0.5153	1.0475	0.5552	0.5857	0.5899	0.1940
Bi-objective optimization (η and $\bar{E}$)	LINMAP	2.9494	0.4638	0.9430	0.5823	0.5904	0.5735	0.3658
	TOPSIS	2.9402	0.4637	0.9468	0.5817	0.5914	0.5743	0.3589
	Shannon Entropy	2.7944	0.4682	1.0032	0.5702	0.5990	0.5848	0.2598
Bi-objective optimization (η and ${\bar{P}}_{d}$)	LINMAP	2.9257	0.4801	0.9529	0.5804	0.5924	0.5766	0.3479
	TOPSIS	2.8976	0.4821	0.9642	0.5783	0.5946	0.5791	0.3278
	Shannon Entropy	2.6480	0.5153	1.0475	0.5552	0.5857	0.5899	0.1940
Bi-objective optimization ($\bar{E}$ and ${\bar{P}}_{d}$)	LINMAP	2.7431	0.4844	1.0212	0.5654	0.5974	0.5878	0.2301
	TOPSIS	2.7443	0.4833	1.0209	0.5655	0.5975	0.5877	0.2307
	Shannon Entropy	2.6481	0.5153	1.0475	0.5552	0.5857	0.5899	0.1940
Maximum $\bar{P}$	——	2.1467	0.4827	1.1353	0.4841	0.3945	0.5403	0.3560
Maximum η	——	3.4435	0.4414	0.7043	0.6002	0.4698	0.4840	0.7693
Maximum $\bar{E}$	——	2.7945	0.4682	1.0032	0.5702	0.5990	0.5848	0.2599
Maximum ${\bar{P}}_{d}$	——	2.6481	0.5153	1.0475	0.5552	0.5857	0.5899	0.1940
Positive ideal point		——	——	1.1353	0.6002	0.5990	0.5899	——
Negative ideal point		——	——	0.7043	0.4840	0.3941	0.4840	——

**Table 2:** Results of single-, bi-, tri- and quadru-objective optimizations.

Optimization Objective	Decision-Making Approaches	Optimization Variables		Optimization Objectives				Deviation Index
		x	u	${\bar{P}}_{s}$	$\eta_{s}$	$\bar{E}$	${\bar{P}}_{d}$	D
Quadru-objective optimization ($\bar{P}$, η, $\bar{E}$ and ${\bar{P}}_{d}$)	LINMAP	2.6197	0.4689	1.0552	0.5507	0.5776	0.5836	0.1767
	TOPSIS	2.6197	0.4689	1.0552	0.5507	0.5776	0.5836	0.1767
	Shannon Entropy	2.6437	0.5146	1.0439	0.5524	0.5760	0.5859	0.1950
Tri-objective optimization ($\bar{P}$, η and $\bar{E}$)	LINMAP	2.6159	0.4791	1.0565	0.5502	0.5768	0.5843	0.1753
	TOPSIS	2.6159	0.4791	1.0565	0.5502	0.5768	0.5843	0.1753
	Shannon Entropy	2.7936	0.4673	0.9985	0.5676	0.5897	0.5807	0.2636
Tri-objective optimization ($\bar{P}$, η and ${\bar{P}}_{d}$)	LINMAP	2.57759	0.4730	1.0676	0.5458	0.5704	0.5830	0.1649
	TOPSIS	2.5591	0.4798	1.0721	0.5438	0.5669	0.5830	0.1623
	Shannon Entropy	2.6435	0.5147	1.0440	0.5524	0.5759	0.5859	0.1949
Tri-objective optimization ($\bar{P}$, $\bar{E}$ and ${\bar{P}}_{d}$)	LINMAP	2.5783	0.4775	1.0670	0.5460	0.5707	0.5834	0.1654
	TOPSIS	2.5896	0.4719	1.0638	0.5474	0.5729	0.5832	0.1679
	Shannon Entropy	2.6436	0.5147	1.0440	0.5524	0.5759	0.5859	0.1949
Tri-objective optimization (η, $\bar{E}$ and ${\bar{P}}_{d}$)	LINMAP	2.8927	0.4843	0.9610	0.5752	0.5855	0.5752	0.6700
	TOPSIS	2.8927	0.4843	0.9610	0.5752	0.5855	0.5752	0.6700
	Shannon Entropy	2.6436	0.5147	1.0440	0.5524	0.5759	0.5859	0.1949
Bi-objective optimization ($\bar{P}$ and η)	LINMAP	2.5736	0.4747	1.0682	0.5455	0.5699	0.5830	0.1645
	TOPSIS	2.5364	0.4753	1.0778	0.5412	0.5623	0.5817	0.1603
	Shannon Entropy	2.1451	0.4823	1.1307	0.4820	0.3848	0.5369	0.3601
Bi-objective optimization ($\bar{P}$ and $\bar{E}$)	LINMAP	2.5778	0.4712	1.0670	0.5461	0.5708	0.5829	0.1654
	TOPSIS	2.5872	0.4738	1.0645	0.5471	0.5724	0.5833	0.1673
	Shannon Entropy	2.7937	0.4672	0.9985	0.5676	0.5897	0.5807	0.2637
Bi-objective optimization ($\bar{P}$ and ${\bar{P}}_{d}$)	LINMAP	2.3995	0.4939	1.1065	0.5231	0.5207	0.5739	0.1942
	TOPSIS	2.4022	0.5005	1.1053	0.5233	0.5209	0.5746	0.1944
	Shannon Entropy	2.6436	0.5146	1.0440	0.5524	0.5760	0.5859	0.1949
Bi-objective optimization (η and $\bar{E}$)	LINMAP	2.9377	0.4643	0.9429	0.5787	0.5823	0.5703	0.3626
	TOPSIS	2.9377	0.4643	0.9429	0.5787	0.5823	0.5703	0.3626
	Shannon Entropy	2.7934	0.4672	0.9986	0.5676	0.5897	0.5807	0.2635
Bi-objective optimization (η and ${\bar{P}}_{d}$)	LINMAP	2.9256	0.4864	0.9476	0.5775	0.5826	0.5724	0.3543
	TOPSIS	2.9102	0.4807	0.9541	0.5766	0.5844	0.5736	0.3422
	Shannon Entropy	2.6436	0.5146	1.0440	0.5524	0.5759	0.5859	0.1949
Bi-objective optimization ($\bar{E}$ and ${\bar{P}}_{d}$)	LINMAP	2.7410	0.4824	1.0170	0.5627	0.5882	0.5836	0.2325
	TOPSIS	2.7425	0.4812	1.0166	0.5629	0.5883	0.5835	0.2333
	Shannon Entropy	2.6437	0.5147	1.0440	0.5524	0.5759	0.5859	0.1949
Maximum $\bar{P}$	——	2.1453	0.4822	1.1307	0.4821	0.3850	0.5370	0.3600
Maximum η	——	3.4266	0.4406	0.7082	0.5964	0.4664	0.4835	0.7630
Maximum $\bar{E}$	——	2.7937	0.4673	0.9985	0.5676	0.5897	0.5807	0.2637
Maximum ${\bar{P}}_{d}$	——	2.6436	0.5146	1.0440	0.5524	0.5759	0.5859	0.1949
Positive ideal point		——	——	1.1307	0.5964	0.5897	0.5859	——
Negative ideal point		——	——	0.7082	0.4820	0.3849	0.4835	——

## Data Availability

Not applicable.

## Nomenclature

B	Magnetic field, T
$C_{wf}$	Mass flow rate times the specific heat, kW/K
D	Deviation index
E	Ecological function, W
e	Electric field, N/C
$E_{H}$	Effectiveness of the heat exchanger on the high-temperature side
$E_{L}$	Effectiveness of the heat exchanger on the low-temperature side
h	Gas enthalpy, J/kg
J	Electric current density, A/m^2^
k	Specific heat ratio
M	Mach number
P	Power output, W
p	Pressure, Pa
$P_{d}$	Power density, W/m^3^
$Q_{H}$	Heat release rate, W
$Q_{L}$	Heat absorption rate, W
T	Temperature, K
$U_{T}$	Total heat exchanger, kW/K
$U_{H}$	High temperature side heat exchanger, kW/K
$U_{L}$	Low temperature side heat exchanger, kW/K
u	Heat exchanger thermal conductance distribution
V	Specific volume, m^3^/kg
v	Gas velocity, m/s
x	Isentropic temperature ratio
Z	Axial direction of the generator
Greek symbols	
η	Thermal efficiency
$\eta_{c}$	Compression efficiency
$\eta_{e}$	Generator efficiency
ρ	Gas density, kg/m^3^
σ	Entropy generation rate, W/K
τ	Temperature ratio of the circulating heat reservoirs
Subscripts	
H	High temperature heat source
L	Low temperature heat sink
opt	Optimal
0	Environment
1−4	State points
Superscripts	
−	Dimensionless

## Abbreviations

CGV	Constant gas velocity
CMN	Constant Mach number
FTT	Finite time thermodynamics
HEX	Heat exchanger
MHD	Magnetohydrodynamic
MOO	Multi-objective optimization
OF	Objective function
